# Sclera color enhances gaze perception in humans

**DOI:** 10.1371/journal.pone.0228275

**Published:** 2020-02-27

**Authors:** Jessica L. Yorzinski, Jacob Miller

**Affiliations:** 1 Department of Ecology and Conservation Biology, Texas A&M University, College Station, Texas, United States of America; 2 Department of Anthropology, Texas A&M University, College Station, Texas, United States of America; Birkbeck University of London, UNITED KINGDOM

## Abstract

Gaze perception is an essential behavior that allows individuals to determine where others are directing their attention but we know relatively little about the ways in which eye morphology influences it. We therefore tested whether eyes with conspicuous morphology have evolved to facilitate gaze perception. During a visual search task, we recorded the eye movements of human participants (*Homo sapiens*) as they searched for faces with directed gaze within arrays of faces with averted gaze or the reverse; the faces were large and upright, small and upright, or large and inverted. The faces had sclera that were conspicuous (white or colored lighter than the iris color) or inconspicuous (colored the same or darker than the iris color). We found that participants were fastest and most accurate in finding the faces with conspicuous sclera versus inconspicuous sclera. Our results demonstrate that eyes with conspicuous morphology facilitate gaze perception in humans.

## Introduction

Many species strongly rely on visual communication to learn about their physical and social environments. They can gather information about events and objects as well as uncover the emotional and intentional states of others [1]. Gaze perception—the ability to determine where other individuals are directing their overt attention—plays a fundamental role in this process by enabling individuals to monitor the gaze of others, including assessments of whether others are directing their gaze toward or away from them [2–5]. Despite the importance of gaze perception and its clear adaptive value, we know relatively little about the ways in which eye morphology influences it.

The gaze enhancement hypothesis [6] suggests that eye morphology influences gaze perception. It predicts that eyes with conspicuous morphology have evolved to facilitate gaze perception while eyes with camouflaged morphology have evolved to hide gaze perception.

Previous work has provided some support for the gaze enhancement hypothesis in humans [7–14]. Adults and children show a preference for stuffed animals with eyes that have a white sclera versus those that do not [13]. Furthermore, adult humans are more accurate in evaluating gaze direction when the irises are darker than the sclera (black irises, white pupils, and white sclera) but not the polar reverse (white irises, black pupils, and black sclera; [7]). Similarly, infants prefer looking at faces with white sclera and black irises/pupils but not the polar reverse [12]. Additional studies have also found that sclera luminance impacts gaze perception [8–11,14]. These studies provide growing evidence to suggest that sclera color is important for gaze perception in humans.

Among primates, humans have especially large sclera that are white [6]. In contrast, the sclera of most other nonhuman primates are less exposed and often pigmented. In fact, of 81 nonhuman primate species sampled, 99% had sclera that closely matched the iris color while only 1% had sclera that was darker than the iris. We are unaware of any previous studies in humans that have tested whether white sclera, rather than sclera that match the natural iris color (as seen in most other primate species; [6]), is necessary for gaze perception. It is possible that the contrast between the black pupil and iris color is sufficient for gaze perception [5]. Previous studies examining the impact of sclera color on gaze perception in humans have not manipulated sclera color to resemble that observed in most other primate species (i.e., sclera color matched to the iris color).

We therefore investigated the relationship between gaze perception and sclera color in humans using sclera colors that resemble those found in most other primate species. In particular, we presented participants with digital faces exhibiting naturally-colored sclera (white) or exhibiting sclera that matched the color of the faces’ irises (similar to the 99% of primate species exhibiting this sclera-iris color scheme; [6]). We recorded the eye movements of human participants as they searched for faces (naturally-colored sclera or sclera that matched the iris color) with directed gaze within arrays of faces with averted gaze or vise versa. This visual search task simulates a situation in which participants must rapidly detect gaze direction among a group of faces. We presented them with faces that were large and upright, small and upright, or large and inverted. The large and upright faces simulated viewing faces in up-close interactions while the small and upright faces simulated viewing faces at a distance. The large and inverted faces preserved low-level visual properties (such as contrast and luminance) but disrupted facial configuration [15]. In addition, we also presented participants with digital faces exhibiting sclera that were darker than the color of the faces’ irises (similar to the 1% of primate species exhibiting this sclera-iris color scheme; [6]) as well as digital faces exhibiting sclera that were lighter than the color of the faces’ irises.

We tested the hypothesis that eyes with conspicuous morphology (white sclera or sclera colored lighter than the iris color) have evolved to facilitate gaze perception while eyes with more camouflaged morphology (sclera colored similar to the iris color or darker) have evolved to hide gaze perception [6]. We predicted that participants would be faster to detect the gaze of upright faces with conspicuous versus inconspicuous morphology because the faces with conspicuous gaze have higher contrast. Furthermore, we predicted this effect would be magnified in the large versus small upright faces because gaze is more difficult to detect at a distance [16]. Lastly, we predicted that participants would be faster to detect the gaze of faces with conspicuous versus inconspicuous morphology even when the faces were inverted and therefore had disrupted facial configurations.

## Materials and methods

### Participants

Adult humans participated in this study at Texas A&M University from September 2016 through December 2018. They were of European heritage, between the ages of 18 and 30 years old, and had corrected-to-normal or normal vision. Flyers and e-mails were used to recruit participants. The participants were told that they would be participating in a study that explored gaze detection. They earned $10 for their participation in the ‘search task’ and ‘search keypad task’ experiments and $20 for their participation in the ‘search efficiency task’ experiment (see below). Ninety participants (53 women and 37 men; mean age ± SD: 20.6 ± 2.6 years old) participated in the ‘search task’ (30 participants in each of the three treatments), ninety participants (49 women and 51 men; mean age ± SD: 20.6 ± 2.8 years old) participated in the ‘search keypad task’ (30 participants in each of the three treatments), and sixty participants (30 women and 30 men; mean age ± SD: 19.8 ± 2.4 years old) participated in the ‘search efficiency task.’ The Institutional Review Board of Texas A&M University (#2016-0575D) approved this study. Informed consent was obtained from all participants.

### Equipment

The eye movements of participants were recorded with a Tobii Pro screen-based eye-tracker (X2-60; Tobii Technology, Inc., Danderyd, Sweden; accuracy: 0.4 degrees; data rate: 60 Hz; binocular tracking) that was run by a laptop computer (Dell Mobile Precision 7510) and displayed on a 25” monitor (Dell UltraSharp UP2516D, 2560 x 1440 pixels; Dell Computer Corporation, Round Rock, TX) using custom eye-tracking software (Tobii Studio 3.4, Tobii Technology). The eye-tracker noninvasively monitored the eye movements of participants and recorded the precise location of where they were looking. The participants were positioned approximately 60 cm from the screen and rested their chins in a chin cup (UHCOTech HeadSpot) to minimize head movements. To minimize the possibility that the participants were actively thinking about their eye movement patterns, participants were told that we were measuring the size of their pupils but were not told that their eye movements were being monitored until after they completed the trial. For the ‘search task’ and ‘search keypad task’ study, the luminance of the monitor (sensor positioned in the middle of the monitor and directed toward the screen) displaying a white background was 190 cd/m2, and the illuminance of the testing room (sensor positioned in the middle of the monitor and directed toward the participant) was 250 lux (Spectra Cine PhoRad Meter, SC-820, Burbank, California). For the ‘search efficiency task’ study, the luminance of the monitor (sensor positioned in the middle of the monitor and directed toward the screen) displaying a white background was also 190 cd/m2, but the illuminance of the testing room (sensor positioned in the middle of the monitor and directed toward the participant) was 30 lux (Spectra Cine PhoRad Meter, SC-820, Burbank, California). The ‘search task’ and ‘search keypad task’ studies were conducted in one location while the ‘search efficiency task’ was conducted in another location (the studies were conducted in different locations due to logistical reasons beyond the researchers’ control). The eye-tracker was calibrated (5 points) before each trial began. We used the Tobii Velocity-Threshold Identification filter (I-VT filter; gap fill-in: 75 ms; eye selection: average; velocity calculator window: 20 ms; I-VT classifier threshold: 30 degrees/s; merge adjacent time: 75 ms; merge adjacent angle: 0.5 degrees) to classify fixations and saccades. This filter classifies eye movements as fixations or saccades based upon the velocity of eye movements. Eye movements below and above the velocity threshold (30 degrees/s, in this study) are classified as fixations and saccades, respectively. Eye-tracking data consisted of coordinates of where participants were known to be looking during each sampling point.

### Experimental stimuli

The experimental stimuli were obtained from a face database (Oslo Face Database) in which participants exhibited a neutral expression with their head facing directly toward the camera and their eyes either direct or averted [17]. Because the stimuli were taken from a face database, the people in the stimuli were likely unfamiliar to the participants. The photographs were manipulated using Adobe Photoshop (Adobe Systems, San Jose, California). We removed all corneal reflections by erasing them and using adjacent colors to fill the space.

**Fig 1:**
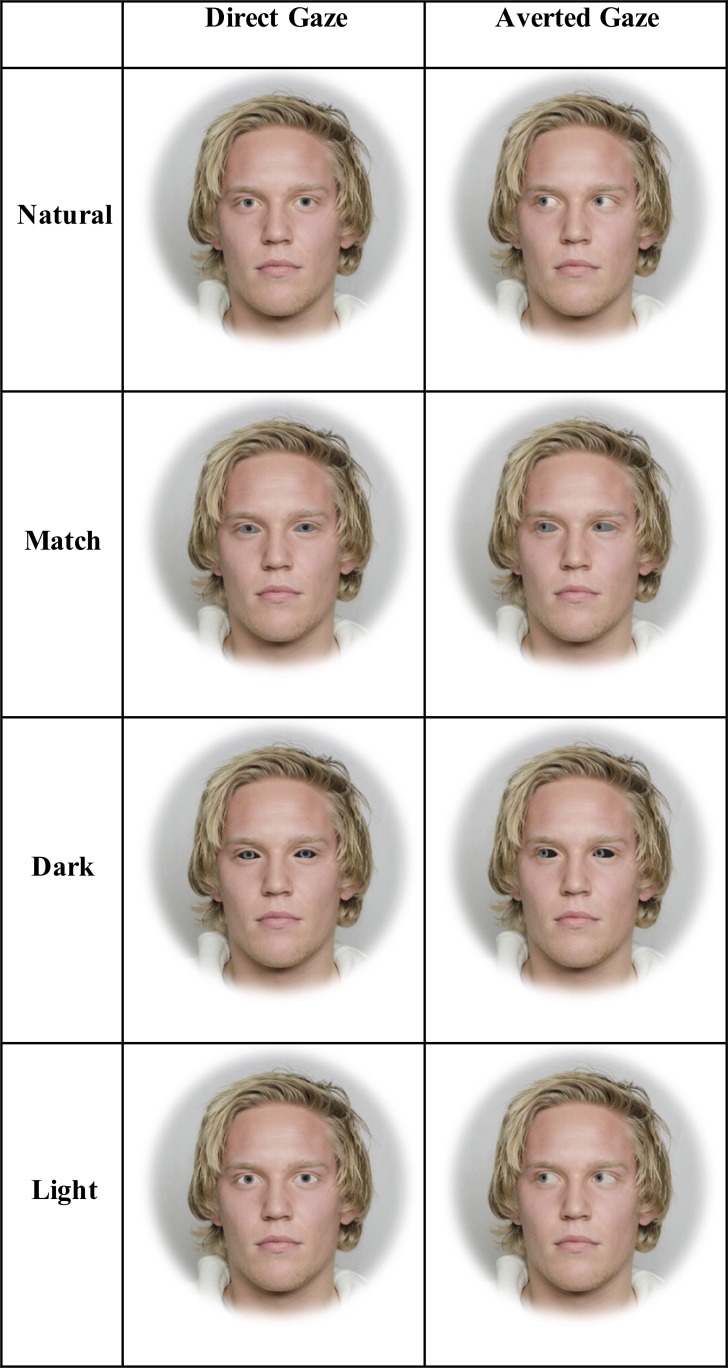
Example stimuli for the match, dark and light treatments when gaze is direct or averted for a face with light irises. The individual in this manuscript has given written informed consent (as outlined in PLOS consent form) to publish these case details [17].

We created three treatments with three blocks of faces each with four sets (Fig 1). The three treatments included modifying the faces such that the sclera matched the color of the iris (‘match’), was lighter than the color of the iris (‘light’), or was darker than the color of the iris (‘dark’). In the ‘match’ treatment, the color of the sclera matched the mean color of the iris. In the ‘light’ and ‘dark’ treatment, the color of the sclera was 75% lighter and 75% darker than the iris, respectively. The ‘match’ sclera colors were created using the average RGB values of the iris; ‘dark’ and ‘light’ sclera colors were created by multiplicatively scaling the average RGB values of the iris by 0.25 and 1.75, respectively. The modified sclera colors that we based on the RGB color space are similar to those that would be generated by the CIELAB color space, a color space that is modelled on the human visual system and designed to be perceptually uniform [18]. In particular, if the sclera colors had been generated using the CIELAB color space rather than the RGB color space, the average difference in sclera colors between our stimuli (based on the RGB color space) and stimuli using the CIELAB color space would have been minimal (match sclera: L*: 0, a*: 0, b*: 0; light: L*: 3, a*: -3, b*: -8; dark: L*: 2, a*: 3, b*: 10).

The three blocks included faces that were upright and subtended 5.7° (‘large and upright’), faces that were upright and subtended 2.85° (‘small and upright’), and faces that were inverted and subtended 5.7° (‘large and inverted’). Within each of the three blocks, four sets of 8-array photographs were shown: (1) face with a direct gaze and natural color within an array of faces with averted gaze and natural color (‘Target Directed Natural’), (2) face with a direct gaze and modified sclera color within an array of faces with averted gaze and modified sclera color (‘Target Directed Modified’), (3) face with an averted gaze and natural color within an array of faces with direct gaze and natural color (‘Target Averted Natural’), and (4) face with an averted gaze and modified sclera color within an array of faces with direct gaze and modified sclera color (‘Target Averted Modified’; Fig 1 and S1A Fig). Within each set, 40 different faces were shown; half of these faces had light-colored irises (subjectively categorized as blue or green; a*: less than two; b*: less than eight; mean ± SE L*: 48.8 ± 0.99) and the other half of these faces had dark-colored irises (subjectively categorized as brown; a*: greater than four; b*: greater than seventeen; mean ± SE L*: 35.5 ± 1.4).

### Experimental procedure

The experimenter first asked participants to perform a practice trial so they could become familiar with the procedure. In the practice trial, the participants were shown arrays of photographs of domestic cats (*Felis catus*). In each array, seven of the cat faces had eyes that were directed and one had eyes that were averted. Participants were instructed to search for the cat face that had its eyes averted as quickly and accurately as possible and then press the space bar. They were also shown arrays of photographs of cats with the opposite arrangement: seven of the cat faces had eyes that were averted and one had eyes that were directed. They were instructed to search for the cat face that had its eyes directed as quickly and accurately as possible and then press the space bar. After they pressed the space bar, the next array appeared.

After completing the practice trial, participants performed the experimental task (‘search task’). The participants were instructed to search for the directed gaze within the arrays of one face with a directed gaze and seven faces with averted gaze (Target Directed Natural & Target Directed Modified) and then press the space bar; or they were instructed to search for the averted gaze within the arrays of one face with an averted gaze and seven faces with directed gaze (Target Averted Natural & Target Averted Modified) and then press the space bar. After they pressed the space bar, the next array appeared. This visual search paradigm is commonly used in studies investigating attention [19–23]. A given participant completed only one treatment (match, light, or dark; randomized across participants) to minimize any effects of participant fatigue. The blocks and sets were randomized within participants.

Because participants did not necessarily identify the correct target (i.e., they may have pressed the space bar but incorrectly identified the target), we ran an additional experimental task (‘search keypad task’) that was similar to the above task except that participants indicated their responses using a custom keypad (model: CP24-USBHID; Genovation, Irvine, CA). The keypad had eight keys that were arranged in the same configuration as the experimental stimuli (a 3 x 3 grid with no key in the middle). In the practice trial and experimental task, the participants pressed the key that corresponded to the stimuli that they identified as the correct target.

Lastly, to examine search efficiency, we ran another experimental task (‘search efficiency task’) that was similar to the ‘search keypad task’ except that participants were presented with arrays consisting of 4 photographs as well as 8 photographs (S1 Fig). Participants only completed the match treatment for this task. Each participant viewed three blocks with four sets each for both 4-array photographs and 8-array photographs.

### Measurements and statistical analysis

Using a customized MATLAB program, we drew rectangular regions of interest (ROIs) around each face. For each fixation coordinate, we determined which ROI it fell within to determine whether the participant was looking at the target face, distractor faces, or neither the target nor distractor faces. We calculated the amount of time that elapsed before participants fixated on the target (Latency to Fixate Target) and the amount of time that elapsed between the participants fixating the correct target and manually responding by pressing a key to indicate they detected the correct target (Latency to Press Key After Fixate Target). For each participant, we calculated the mean value of the metrics within each of the four sets (Target Directed Natural, Target Directed Modified, Target Averted Natural, and Target Averted Modified) of the three blocks (large, small, and inverted) for each treatment (match, light, and dark). In arrays where the data indicated a participant never fixated the target, it was not possible to determine whether the participants did not fixate the target (and therefore did not correctly perform the task) or whether the eye-tracker failed to record the participants’ gaze when they were fixating the target. We therefore excluded a given matrix from the analysis if a participant’s fixations never fell within the target or if more than 10% of the gaze data was missing (5.9%, 9.6%, and 4.6% of matrices were excluded in the ‘search task,’ ‘search keypad task,’ and ‘search efficiency task,’ respectively).

We analyzed our data using linear mixed-effects models with repeated measures in SAS (PROC MIXED; unstructured covariance structure; Version 9.4; SAS Institute Inc., Cary, NC). The dependent variable was the latency to fixate the target face. The independent variables were the block (large, small, or inverted), set (Target Directed Natural, Target Directed Modified, Target Averted Natural, and Target Averted Modified), treatment (match, light, and dark), iris color (light or dark) and their interactions, as well as the block order (order in which the blocks were displayed for each participant), age and gender of the participants. Participant identity was included within the models to account for repeated measures. We performed *a priori* contrasts to compare the latency to detect the target face between the modified sclera (match, light, or dark) and the natural sclera; we performed 18 comparisons and used the false discovery rate correction [24] to evaluate statistical significance. We performed similar repeated-measures mixed linear models using the latency to indicate their response (key press) after fixating the target as the dependent variable. We ran separate models for the ‘search task’ and ‘search keypad task’ experiments. In addition, we performed a similar generalized linear mixed model (PROC GLIMMIX; Poisson distribution) using the percentage of correct responses as the dependent variable in the ‘search keypad task.’

Finally, we performed a linear mixed-effects model with repeated measures to examine search efficiency. The model was the same as the linear mixed-effects models above except that array size (four or eight faces) was included as an additional independent variable, treatment was excluded as an independent variable (participants only completed the match treatment), a variance component structure was used (the structure that resulted in the best model fit), and we performed 12 *a priori* comparisons. We also ran a similar linear mixed-effects model using slope (the slope of each set in the four versus eight face arrays; “search efficiency”; [25]) as the dependent variable.

## Results

**Fig 2:**
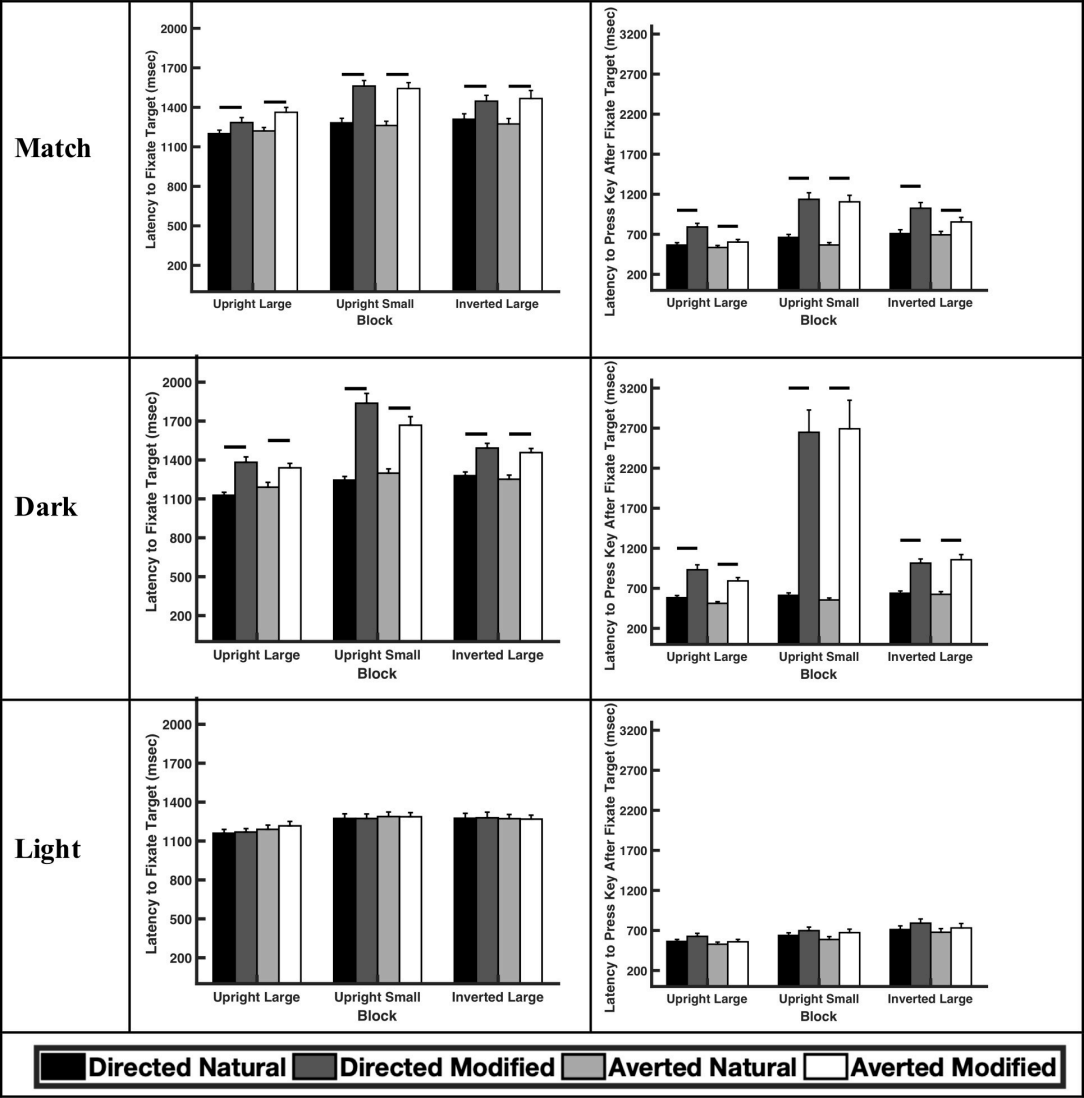
The latency to initially fixate the target and latency to indicate choice (via key press) after initially fixating the target for large and upright faces, small and upright faces, and large and inverted faces for the match, dark, and light treatment in the ‘search task’. Horizontal lines indicate planned comparisons that were statistically significant.

Sclera color influenced the amount of time participants spent searching for the target faces (F_2,80_ = 6.35, p = 0.0028; Table 1: Latency to Fixate Target; Fig 2). Regardless of gaze direction, participants spent more time searching for faces with sclera that matched the iris color (large and upright, directed: F_1,80_ = 2.76, p = 0.0071; large and upright, averted: F_1,80_ = 4.66, p<0.0001) or were darker than the iris color (large and upright, directed: F_1,80_ = 8.48, p<0.0001; large and upright, averted: F_1,80_ = 5.22, p<0.0001) than they did for faces with natural sclera when the faces were relatively large (subtending 5.7°). Similarly, when the faces were relatively small (subtending 2.85°), participants were also slower to find the faces with sclera that matched the iris color (large and upright, directed: F_1,80_ = 5.93, p<0.0001; large and upright, averted: F_1,80_ = 5.76, p<0.0001) or were darker than the iris color (large and upright, directed: F_1,80_ = 12.65, p<0.0001; large and upright, averted: F_1,80_ = 7.66, p<0.0001) compared to faces with natural sclera. These effects were most pronounced when participants were searching for faces in which the sclera were darker than the iris color. For example, when the faces were upright and small, participants were 1.22x and 1.48x slower in searching for the faces with directed gaze when the sclera matched the iris color or was darker than the iris color, respectively, compared to when the sclera were naturally-colored. Even when the faces were inverted, participants still spent more time searching for the faces with sclera that matched the iris color (large and upright, directed: F_1,80_ = 3.88, p = 0.0002; large and upright, averted: F_1,80_ = 5.35, p<0.0001) or were darker than the iris color (large and upright, directed: F_1,80_ = 5.87, p<0.0001; large and upright, averted: F_1,80_ = 5.77, p<0.0001) than they did for faces with natural sclera. When the sclera were lighter than the iris color, participants spent a similar amount of time searching for those faces compared to faces with natural sclera (large and upright, directed: F_1,80_ = 0.30, p = 0.76; large and upright, averted: F_1,80_ = 0.82, p = 0.41). Participants also took more time searching for faces with dark versus light iris colors (F_1,80_ = 23.88, p<0.0001).

**Table 1:** The effect of block, set, treatment, iris color, block order, age and gender on the latency to fixate the target and latency to press a key after fixating the target for the ‘search task’.

	Numerator df, Denominator df	Latency to Fixate Target	Latency to Press Key After Fixate Target
**Overall model**			
Block	2, 80	88.65 (<0.0001)*	60.63 (<0.0001)*
Set	3, 80	59.02 (<0.0001)*	59.44 (<0.0001)*
Treatment	2, 80	6.35 (0.0028)*	18.73 (<0.0001)*
Iris Color	1, 80	23.88 (<0.0001)*	66.22 (<0.0001)*
Block*Set	6, 80	9.33 (<0.0001)*	12.88 (<0.0001)*
Block*Treatment	4, 80	5.90 (0.0003)*	16.67 (<0.0001)*
Block*Iris Color	2, 80	6.90 (0.0017)*	13.78 (<0.0001)*
Set*Treatment	6, 80	17.85 (<0.0001)*	18.65 (<0.0001)*
Set* Iris Color	3, 80	4.81 (0.0039)*	28.50 (<0.0001)*
Treatment* Iris Color	2, 80	2.48 (0.090)	19.25 (<0.0001)*
Block*Set*Treatment	12, 80	3.68 (0.0002)*	7.35 (<0.0001)*
Block*Set*Iris Color	6, 80	1.86 (0.098)	6.69 (<0.0001)*
Block*Treatment*Iris Color	4, 80	2.10 (0.089)	4.50 (0.0025)*
Set*Treatment*Iris Color	6, 80	1.54 (0.17)	7.73 (<0.0001)*
Block*Set*Treatment* Iris Color	12, 80	1.48 (0.15)	3.41 (0.0005)*
Block Order	5, 80	1.00 (0.43)	2.33 (0.0496)*
Age	1, 80	0.11 (0.74)	0.00 (0.96)
Gender	1, 80	3.57 (0.063)	2.00 (0.16)
**Comparisons**			
Match: Large and Upright			
Target Directed Natural vs. Directed Modified	1, 80	2.76 (0.0071)*	5.77 (<0.0001)*
Target Averted Natural vs. Averted Modified	1, 80	4.66 (<0.0001)*	2.17 (0.033)*
Match: Small and Upright			
Target Directed Natural vs. Directed Modified	1, 80	5.93 (<0.0001)*	3.16 (0.0022)*
Target Averted Natural vs. Averted Modified	1, 80	5.76 (<0.0001)*	2.48 (0.015)*
Match: Large and Inverted			
Target Directed Natural vs. Directed Modified	1, 80	3.88 (0.0002)*	7.60 (<0.0001)*
Target Averted Natural vs. Averted Modified	1, 80	5.35 (<0.0001)*	3.09 (0.0027)*
Dark: Large and Upright			
Target Directed Natural vs. Directed Modified	1, 80	8.48 (<0.0001)*	8.96 (<0.0001)*
Target Averted Natural vs. Averted Modified	1, 80	5.22 (<0.0001)*	9.16 (<0.0001)*
Dark: Small and Upright			
Target Directed Natural vs. Directed Modified	1, 80	12.65 (<0.0001)*	13.31 (<0.0001)*
Target Averted Natural vs. Averted Modified	1, 80	7.66 (<0.0001)*	9.92 (<0.0001)*
Dark: Large and Inverted			
Target Directed Natural vs. Directed Modified	1, 80	5.87 (<0.0001)*	8.91 (<0.0001)*
Target Averted Natural vs. Averted Modified	1, 80	5.77 (<0.0001)*	8.25 (<0.0001)*
Light: Large and Upright			
Target Directed Natural vs. Directed Modified	1, 80	0.30 (0.76)	1.62 (0.11)
Target Averted Natural vs. Averted Modified	1, 80	0.82 (0.41)	0.98 (0.33)
Light: Small and Upright			
Target Directed Natural vs. Directed Modified	1, 80	0.01 (0.99)	0.39 (0.70)
Target Averted Natural vs. Averted Modified	1, 80	0.05 (0.96)	0.39 (0.70)
Light: Large and Inverted			
Target Directed Natural vs. Directed Modified	1, 80	0.11 (0.92)	1.92 (0.058)
Target Averted Natural vs. Averted Modified	1, 80	0.27 (0.79)	1.00 (0.32)

F values are displayed for the overall model and t values are displayed for the comparisons; p-values are indicated in parentheses.

*Statistically significant

The results were similar in regards to the latency of participants to indicate their responses after initially fixating on the correct target (Table 1: Latency to Press Key After Fixate Target; Fig 2). Participants were slower to indicate their responses for faces with sclera that matched the iris color (large and upright, directed: F_1,80_ = 5.77, p<0.0001; large and upright, averted: F_1,80_ = 2.17, p = 0.033) or were darker than the iris color (large and upright, directed: F_1,80_ = 8.96, p<0.0001; large and upright, averted: F_1,80_ = 9.16, p<0.0001) than they were for faces with natural sclera. There was no difference in the latency to indicate their responses for faces with sclera that were lighter than the iris color compared to faces with natural sclera (large and upright, directed: F_1,80_ = 1.62, p = 0.11; large and upright, averted: F_1,80_ = 0.98, p = 0.33). The results were also similar when the participants completed the same task but also manually indicated (via a keypad) which face was the correct target (S1 Table).

Overall, participants were accurate in selecting faces that were the correct target (mean accuracy above 98%; S2 Table; S2 Fig). However, their accuracy in selecting the correct target varied based on sclera color and face size (F_12,519_ = 13.07, p<0.0001). Their accuracy was lowest (mean accuracy less than 90%) for the faces that were small and had sclera that were darker than the iris color (directed: F_1,519_ = 12.93, p<0.0001; averted: F_1,519_ = 14.16, p<0.0001). Participants accuracy was higher for faces with light compared to dark irises (F_1,87_ = 31.77, p<0.0001).

As expected, participants spent more time searching for the correct target when there were eight versus four faces within each array (F_1,59_ = 6286.20, p<0.0001; S3 Table; S3 Fig). Their search efficiency for small faces with sclera that matched the iris color was least efficient [25]: the search slopes for small faces with sclera that matched the iris color were steeper than the search slopes for small faces with natural sclera (directed: F_1,354_ = 3.12, p = 0.0020; averted: F_1,354_ = 2.84, p = 0.0047; S4 Table; S3 Fig). The search efficiency for large and upright faces (directed: F_1,354_ = 1.40, p = 0.16; averted: F_1,354_ = 1.18, p = 0.24) as well as inverted faces (directed: F_1,354_ = 1.73, p = 0.085; averted: F_1,354_ = 2.03, p = 0.043) was similar regardless of sclera color.

## Discussion

Our results support the hypothesis that eyes with conspicuous morphology have evolved to facilitate gaze perception. We found that eyes with conspicuous morphology are necessary for rapid gaze perception in humans: adult participants were faster at detecting target faces with conspicuous sclera (white sclera or sclera colored lighter than the iris color) compared to faces with inconspicuous sclera (sclera colored similar to the iris color or darker). These results demonstrate that conspicuous sclera, in contrast to the camouflaged sclera observed in most other primate species [6], are critical for rapid gaze perception in humans.

Human participants rapidly fixated target faces with conspicuous sclera. Regardless of whether participants were searching for the face with directed gaze among faces with averted gaze or the face with averted gaze among faces with directed gaze, they quickly found the target face with conspicuous sclera. In contrast, when the sclera was inconspicuous, participants spent more time searching for those faces compared to the faces with conspicuous sclera. These patterns were similar for the large faces, which simulated close-up interactions, as well as small faces, which simulated distant interactions. They were also similar for the inverted faces, which preserved low-level visual properties (such as luminance) but disrupted facial configuration [26]. Because visual attention can be strongly influenced by bottom-up processes (such as contrast; [27,28]), it is not necessarily surprising that participants were fast to fixate target faces with conspicuous sclera that exhibit high contrast. Importantly, participants were equally rapid at fixating faces with naturally-colored sclera and faces with sclera color that were lighter than the iris color; this demonstrates that digitally manipulating sclera color does not necessarily alter fixation latencies. We also found that participants were quicker to fixate target faces with light versus dark colored irises; future experiments can assess how iris color interacts with sclera color to influence gaze perception.

After participants fixated target faces with conspicuous sclera, they were quick to manually indicate that they had found the target faces (via a key press). However, when the sclera was inconspicuous, participants were much slower to manually indicate that they had found the target faces; their manual response times were especially slow for the small faces with sclera color that were darker than the iris color. These slower manual responses likely indicate that the participants were less certain of their decisions and therefore taking more time to indicate their responses. Their manual response times for faces with naturally-colored sclera and faces with sclera color that were lighter than the iris color were similar, indicating that digitally manipulating sclera color does not necessarily alter manual response latencies.

Participants were highly accurate in selecting the target faces. With the exception of the small faces with sclera that were darker than the iris color, participants were over 98% accurate in finding the correct target face. Therefore, even though participants spent more time searching for the target face when the face had inconspicuous sclera (see above), they were accurate in ultimately identifying the correct face. The only target faces that participants had difficulty in correctly identifying were the small faces with dark sclera color: they were 90% accurate in finding these target faces. Previous work found that humans are only 52% accurate in gaze perception when the sclera are darker than the iris (black sclera, white pupil, and white iris [7]). In contrast, we found that accuracy in gaze perception was over 90% regardless of sclera color. Rather than presenting stimuli in which the sclera and iris color were polar opposites [7], our stimuli only adjusted sclera color (rather than simultaneously adjusting sclera, pupil and iris color) and we did so relative to the natural iris color. Given that most primate species have sclera that closely match their iris color [6], our stimuli in which the sclera color matched the iris color closely simulated the eye morphology of these other primate species. Furthermore, our study used a gaze search task while previous work used a gaze discrimination task, which could also account for differences in accuracy levels.

Surprisingly, search efficiency was generally similar for faces with naturally-colored sclera and faces with sclera that matched the iris color. When the faces were large and upright as well as large and inverted, participants were equally efficient at searching for these faces. However, participants were less efficient at searching for faces with sclera that matched the iris color versus naturally-colored sclera when the faces were small, suggesting that sclera color in gaze perception may be especially important during distant interactions. The ability of humans to communicate with each other over large distances is likely critical to many types of interactions. This ability may be especially useful during a hunt when silent communication is critical for a successful outcome [5,29]. Previous work has shown that humans are able to reliably detect gaze direction from as far away as 10 meters [30,31] and our results suggest that sclera color contributes to this ability.

Our results support the gaze enhancement hypothesis by demonstrating that eyes with conspicuous morphology facilitate gaze perception. Humans are fastest and most accurate in gaze perception when faces have naturally-colored sclera (or sclera that are lighter than the iris color). When faces are modified so that eye morphology is less conspicuous (sclera that match the iris color or are darker than the iris color), gaze perception is slower. Given that 99% of nonhuman primates have eye morphology that is less conspicuous (with sclera color that closely matches their iris color; [6]), future experiments that examine nonhuman primates’ ability to perceive gaze would be informative. Our results demonstrate that eye morphology plays a critical role in human gaze perception and therefore impacts the evolution of social cognition.

## Supporting information

S1 Table The effect of block, set, treatment, iris color, block order, age and gender on the latency to fixate the target and latency to press a key after fixating the target for the ‘search keypad task’. (DOCX)

S2 Table The effect of block, set, treatment, iris color, block order, age and gender on the percentage of correct responses for the ‘search keypad task’. (DOCX)

S3 Table The effect of block, set, iris color, array size, block order, age and gender on the latency to fixate the target and latency to press a key after fixating the target for the ‘search efficiency task’. (DOCX)

S4 Table The effect of block, set, iris color, block order, age and gender on the slopes of the ‘search efficiency task’. (DOCX)

S1 Fig Example of (A) 8-array and (B) 4-array stimuli used in the search tasks for the Target Averted Natural set (the 4-array stimuli were only used in the ‘search efficiency task’).
(DOCX)

S2 Fig The percentage of correct responses in the ‘search keypad task’ for the match, dark, and light treatment. Horizontal lines indicate planned comparisons that were statistically significant.
(DOCX)

S3 Fig The latency to initially fixate the target for large and upright faces, small and upright faces, and large and inverted faces for the match treatment in the ‘search efficiency task’. (DOCX)
