# Supplementary material for: Sclera color enhances gaze perception in humans
Source: PLoS One. 2020 Feb 27;15(2):e0228275. doi: 10.1371/journal.pone.0228275 (PMC7046275; doi:10.1371/journal.pone.0228275)
Supplement: S1 Table — (DOCX) [file pone.0228275.s001.docx]

**S1 Table**

|  | Numerator df, Denominator df | Latency to Fixate Target | Latency to Press Key After Fixate Target |
| --- | --- | --- | --- |
| **Overall model** |  |  |  |
| Block | 2, 80 | 41.31 (<0.0001)* | 40.30 (<0.0001)* |
| Set | 3, 80 | 51.61 (<0.0001)* | 52.88 (<0.0001)* |
| Treatment | 2, 80 | 20.22 (<0.0001)* | 40.34 (<0.0001)* |
| Iris Color | 1, 80 | 25.63 (<0.0001)* | 159.77 (<0.0001)* |
| Block*Set | 6, 80 | 8.25 (<0.0001)* | 15.04 (<0.0001)* |
| Block*Treatment | 4, 80 | 8.83 (<0.0001)* | 24.37 (<0.0001)* |
| Block*Iris Color | 2, 80 | 4.34 (0.016)* | 23.97 (<0.0001)* |
| Set*Treatment | 6, 80 | 14.53 (<0.0001)* | 24.26 (<0.0001)* |
| Set* Iris Color | 3, 80 | 4.54 (0.0055)* | 53.50 (<0.0001)* |
| Treatment* Iris Color | 2, 80 | 6.96 (0.0016)* | 55.97 (<0.0001)* |
| Block*Set*Treatment | 12, 80 | 4.67 (<0.0001)* | 10.60 (<0.0001)* |
| Block*Set*Iris Color | 6, 80 | 3.82 (0.0021)* | 11.14 (<0.0001)* |
| Block*Treatment*Iris Color | 4, 80 | 1.29 (0.28) | 15.50 (<0.0001)* |
| Set*Treatment*Iris Color | 6, 80 | 2.18 (0.054) | 21.43 (<0.0001)* |
| Block*Set*Treatment* Iris Color | 12, 80 | 1.05 (0.41) | 5.90 (<0.0001)* |
| Block Order | 5, 80 | 7.91 (<0.0001)* | 2.53 (0.036)* |
| Age | 1, 80 | 0.72 (0.40) | 0.11 (0.74) |
| Gender | 1, 80 | 4.84 (0.031)* | 1.06 (0.31) |
| **Comparisons** |  |  |  |
| Match: Large and Upright |  |  |  |
| Target Directed Natural vs. Directed Modified | 1, 80 | 3.27 (0.0015)* | 2.09 (0.039) |
| Target Averted Natural vs. Averted Modified | 1, 80 | 3.39 (0.0011)* | 1.94 (0.056) |
| Match: Small and Upright |  |  |  |
| Target Directed Natural vs. Directed Modified | 1, 80 | 4.55 (<0.0001)* | 2.41 (0.018)* |
| Target Averted Natural vs. Averted Modified | 1, 80 | 4.23 (<0.0001)* | 2.74 (0.0074)* |
| Match: Large and Inverted |  |  |  |
| Target Directed Natural vs. Directed Modified | 1, 80 | 3.65 (0.0004)* | 5.22 (<0.0001)* |
| Target Averted Natural vs. Averted Modified | 1, 80 | 4.15 (<0.0001)* | 3.64 (0.0005)* |
| Dark: Large and Upright |  |  |  |
| Target Directed Natural vs. Directed Modified | 1, 80 | 2.47 (0.015)* | 7.18 (<0.0001)* |
| Target Averted Natural vs. Averted Modified | 1, 80 | 5.77 (<0.0001)* | 8.78 (<0.0001)* |
| Dark: Small and Upright |  |  |  |
| Target Directed Natural vs. Directed Modified | 1, 80 | 10.42 (<0.0001)* | 13.38 (<0.0001)* |
| Target Averted Natural vs. Averted Modified | 1, 80 | 12.17 (<0.0001)* | 14.21 (<0.0001)* |
| Dark: Large and Inverted |  |  |  |
| Target Directed Natural vs. Directed Modified | 1, 80 | 5.11 (<0.0001)* | 7.02 (<0.0001)* |
| Target Averted Natural vs. Averted Modified | 1, 80 | 3.66 (0.0004)* | 5.82 (<0.0001)* |
| Light: Large and Upright |  |  |  |
| Target Directed Natural vs. Directed Modified | 1, 80 | 1.5 (0.14) | 0.13 (0.90) |
| Target Averted Natural vs. Averted Modified | 1, 80 | 0.2 (0.84) | 0.99 (0.32) |
| Light: Small and Upright |  |  |  |
| Target Directed Natural vs. Directed Modified | 1, 80 | 0.5 (0.62) | 0.23 (0.82) |
| Target Averted Natural vs. Averted Modified | 1, 80 | 0.39 (0.70) | 0.68 (0.50) |
| Light: Large and Inverted |  |  |  |
| Target Directed Natural vs. Directed Modified | 1, 80 | 0.15 (0.88) | 1.51 (0.14) |
| Target Averted Natural vs. Averted Modified | 1, 80 | 1.07 (0.29) | 0.44 (0.66) |

F values are displayed for the overall model and t values are displayed for the comparisons; p-values are indicated in parentheses.

*Statistically significant
