# Supplementary material for: Sclera color enhances gaze perception in humans
Source: PLoS One. 2020 Feb 27;15(2):e0228275. doi: 10.1371/journal.pone.0228275 (PMC7046275; doi:10.1371/journal.pone.0228275)
Supplement: S2 Table — (DOCX) [file pone.0228275.s002.docx]

**S2 Table**

|  | Numerator df, Denominator df | Correct Response |
| --- | --- | --- |
| **Overall model** |  |  |
| Block | 2, 174 | 45.73 (<0.0001)* |
| Set | 3, 261 | 21.62 (<0.0001)* |
| Treatment | 2, 80 | 31.09 (<0.0001)* |
| Iris Color | 1, 87 | 31.77 (<0.0001)* |
| Block*Set | 6, 519 | 14.42 (<0.0001)* |
| Block*Treatment | 4, 174 | 35.48 (<0.0001)* |
| Block*Iris Color | 2, 174 | 19.17 (<0.0001)* |
| Set*Treatment | 6, 261 | 13.51 (<0.0001)* |
| Set* Iris Color | 3, 261 | 11.38 (<0.0001)* |
| Treatment* Iris Color | 2, 87 | 14.90 (<0.0001)* |
| Block*Set*Treatment | 12, 519 | 13.07 (<0.0001)* |
| Block*Set*Iris Color | 6, 511 | 6.40 (<0.0001)* |
| Block*Treatment*Iris Color | 4, 174 | 14.63 (<0.0001)* |
| Set*Treatment*Iris Color | 6, 261 | 6.15 (<0.0001)* |
| Block*Set*Treatment* Iris Color | 12, 511 | 5.61 (<0.0001)* |
| Block Order | 5, 80 | 4.01 (0.0027)* |
| Age | 1, 80 | 0.41 (0.53) |
| Gender | 1, 80 | 4.15 (0.0449)* |
| **Comparisons** |  |  |
| Match: Large and Upright |  |  |
| Target Directed Natural vs. Directed Modified | 1, 519 | 0.26 (0.79) |
| Target Averted Natural vs. Averted Modified | 1, 519 | 0.8 (0.43) |
| Match: Small and Upright |  |  |
| Target Directed Natural vs. Directed Modified | 1, 519 | 1.41 (0.16) |
| Target Averted Natural vs. Averted Modified | 1, 519 | 0.98 (0.33) |
| Match: Large and Inverted |  |  |
| Target Directed Natural vs. Directed Modified | 1, 519 | 0.28 (0.78) |
| Target Averted Natural vs. Averted Modified | 1, 519 | 0.23 (0.82) |
| Dark: Large and Upright |  |  |
| Target Directed Natural vs. Directed Modified | 1, 519 | 0.15 (0.88) |
| Target Averted Natural vs. Averted Modified | 1, 519 | 0.55 (0.58) |
| Dark: Small and Upright |  |  |
| Target Directed Natural vs. Directed Modified | 1, 519 | 12.93 (<0.0001)* |
| Target Averted Natural vs. Averted Modified | 1, 519 | 14.16 (<0.0001)* |
| Dark: Large and Inverted |  |  |
| Target Directed Natural vs. Directed Modified | 1, 519 | 1.29 (0.20) |
| Target Averted Natural vs. Averted Modified | 1, 519 | 0.03 (0.98) |
| Light: Large and Upright |  |  |
| Target Directed Natural vs. Directed Modified | 1, 519 | 0.3 (0.76) |
| Target Averted Natural vs. Averted Modified | 1, 519 | 0.67 (0.50) |
| Light: Small and Upright |  |  |
| Target Directed Natural vs. Directed Modified | 1, 519 | 0.54 (0.59) |
| Target Averted Natural vs. Averted Modified | 1, 519 | 0.23 (0.2) |
| Light: Large and Inverted |  |  |
| Target Directed Natural vs. Directed Modified | 1, 519 | 0.91 (0.37) |
| Target Averted Natural vs. Averted Modified | 1, 519 | 0.32 (0.75) |

F values are displayed for the overall model and t values are displayed for the comparisons; p-values are indicated in parentheses.

*Statistically significant
