# Supplementary material for: Sclera color enhances gaze perception in humans
Source: PLoS One. 2020 Feb 27;15(2):e0228275. doi: 10.1371/journal.pone.0228275 (PMC7046275; doi:10.1371/journal.pone.0228275)
Supplement: S3 Table — (DOCX) [file pone.0228275.s003.docx]

**S3 Table**

|  | Numerator df, Denominator df | Latency to Fixate Target |
| --- | --- | --- |
| **Overall model** |  |  |
| Block | 2, 118 | 83.37 (<0.0001)* |
| Set | 3, 177 | 91.94 (<0.0001)* |
| Array Size | 1, 59 | 6286.20 (<0.0001)* |
| Iris Color | 1, 59 | 12.40 (0.0008)* |
| Block*Set | 6, 354 | 5.50 (<0.0001)* |
| Block*Array Size | 2, 118 | 22.17 (<0.0001)* |
| Block*Iris Color | 2, 118 | 0.35 (0.70) |
| Set*Array Size | 3, 177 | 6.29 (0.0004)* |
| Set* Iris Color | 3, 177 | 1.98 (0.12) |
| Array Size* Iris Color | 1, 59 | 2.41 (0.13) |
| Block*Set*Array Size | 6, 354 | 0.64 (0.70) |
| Block*Set*Iris Color | 6, 354 | 0.37 (0.90) |
| Block*Array Size*Iris Color | 2, 118 | 0.06 (0.94) |
| Set*Array Size*Iris Color | 3, 177 | 0.64 (0.59) |
| Block*Set*Array Size* Iris Color | 6, 354 | 0.34 (0.92) |
| Block Order | 15, 42 | 15.72 (<0.0001)* |
| Age | 1, 42 | 9.30 (0.004) |
| Gender | 1, 42 | 79.00 (<0.0001)* |
| **Comparisons** |  |  |
| Large and Upright |  |  |
| 4 vs. 8: Target Directed Natural | 1, 354 | 18.85 (<0.0001)* |
| 4 vs. 8: Target Averted Natural | 1, 354 | 19.98 (<0.0001)* |
| 4 vs. 8: Target Directed Modified | 1, 354 | 20.50 (<0.0001)* |
| 4 vs. 8: Target Averted Modified | 1, 354 | 21.38 (<0.0001)* |
| Small and Upright |  |  |
| 4 vs. 8: Target Directed Natural | 1, 354 | 21.53 (<0.0001)* |
| 4 vs. 8: Target Averted Natural | 1, 354 | 23.06 (<0.0001)* |
| 4 vs. 8: Target Directed Modified | 1, 354 | 25.21 (<0.0001)* |
| 4 vs. 8: Target Averted Modified | 1, 354 | 26.41 (<0.0001)* |
| Large and Inverted |  |  |
| 4 vs. 8: Target Directed Natural | 1, 354 | 23.61 (<0.0001)* |
| 4 vs. 8: Target Averted Natural | 1, 354 | 23.04 (<0.0001)* |
| 4 vs. 8: Target Directed Modified | 1, 354 | 25.65 (<0.0001)* |
| 4 vs. 8: Target Averted Modified | 1, 354 | 25.43 (<0.0001)* |

F values are displayed for the overall model and t values are displayed for the comparisons; p-values are indicated in parentheses.

*Statistically significant
