# Supplementary material for: Sclera color enhances gaze perception in humans
Source: PLoS One. 2020 Feb 27;15(2):e0228275. doi: 10.1371/journal.pone.0228275 (PMC7046275; doi:10.1371/journal.pone.0228275)
Supplement: S4 Table — (DOCX) [file pone.0228275.s004.docx]

**S4 Table**

|  | Numerator df, Denominator df | Slope |
| --- | --- | --- |
| **Overall model** |  |  |
| Block | 2, 118 | 31.83 (<0.0001)* |
| Set | 3, 177 | 9.03 (<0.0001)* |
| Iris Color | 1, 59 | 3.46 (0.068) |
| Block*Set | 6, 354 | 0.92 (0.48) |
| Block*Iris Color | 2, 118 | 0.09 (0.92) |
| Set*Iris Color | 3, 177 | 0.92 (0.43) |
| Block*Set*Iris Color | 6, 354 | 0.48 (0.82) |
| Block Order | 15, 42 | 6.91 (<0.0001)* |
| Age | 1, 42 | 15.88 (0.0003)* |
| Gender | 1, 42 | 19.07 (<0.0001)* |
| **Comparison** |  |  |
| Large and Upright |  |  |
| Target Directed Natural vs. Directed Modified | 1, 354 | 1.40 (0.16) |
| Target Averted Natural vs. Averted Modified | 1, 354 | 1.18 (0.24) |
| Small and Upright |  |  |
| Target Directed Natural vs. Directed Modified | 1, 354 | 3.12 (0.0020)* |
| Target Averted Natural vs. Averted Modified | 1, 354 | 2.84 (0.0047)* |
| Large and Inverted |  |  |
| Target Directed Natural vs. Directed Modified | 1, 354 | 1.73 (0.085) |
| Target Averted Natural vs. Averted Modified | 1, 354 | 2.03 (0.043) |

F values are displayed for the overall model and t values are displayed for the comparisons; p-values are indicated in parentheses.

*Statistically significant
