# Supplementary figures and images for: Sclera color enhances gaze perception in humans
Source: PLoS One. 2020 Feb 27;15(2):e0228275. doi: 10.1371/journal.pone.0228275 (PMC7046275; doi:10.1371/journal.pone.0228275)

**S1 Fig.**

| **A**  **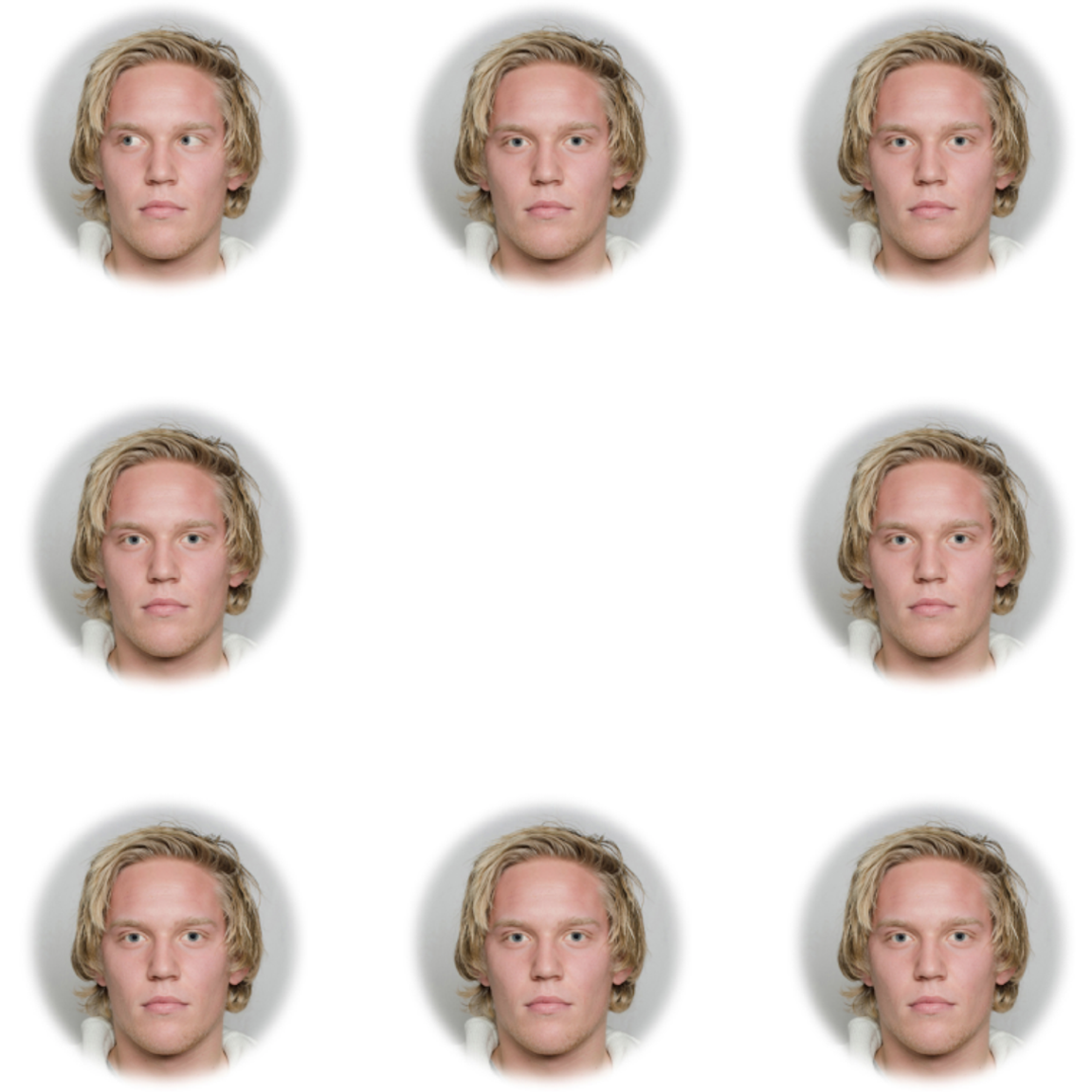** |
| --- |
| **B**  **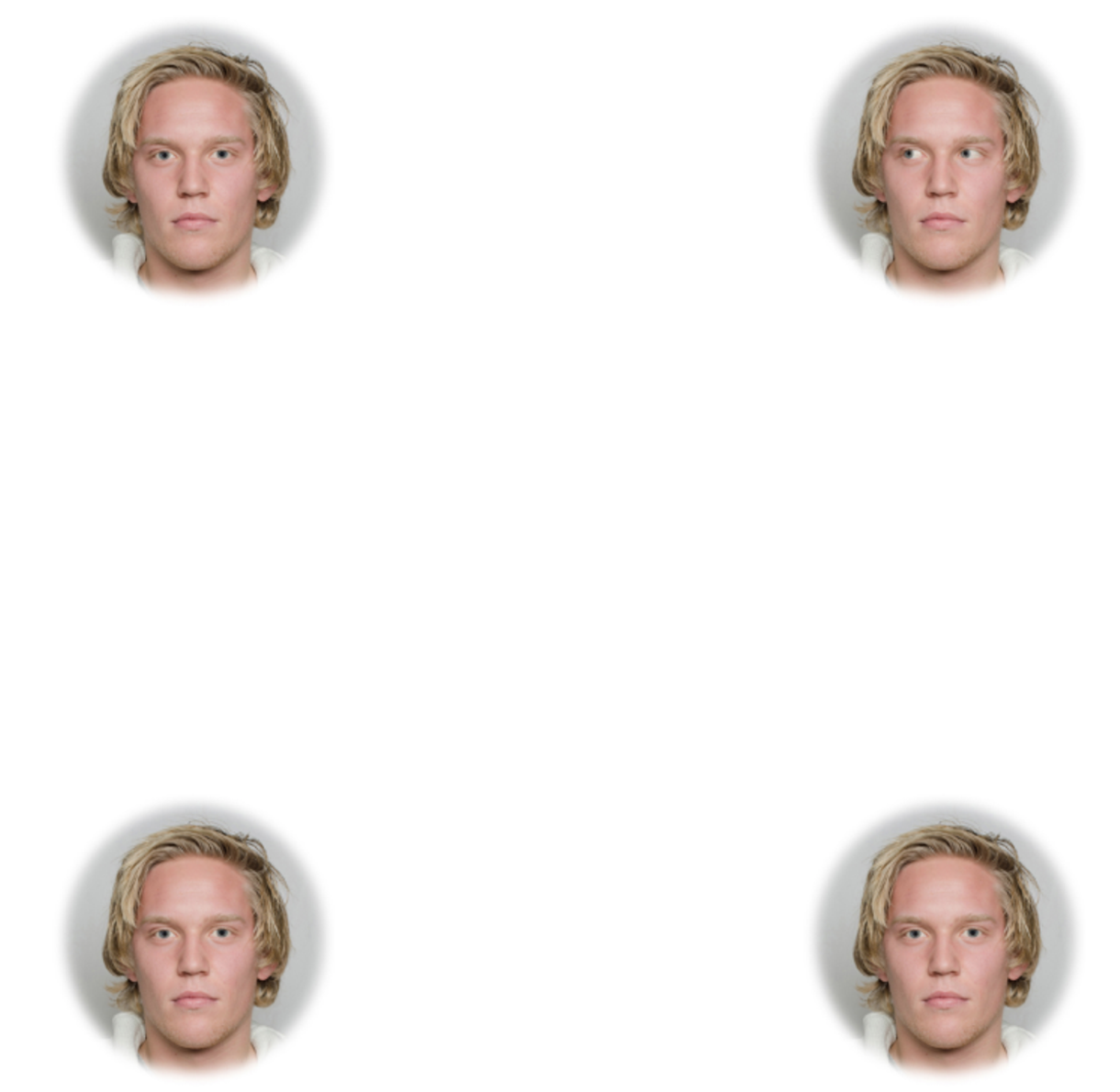** |

Supplement: S1 Fig — Example of (A) 8-array and (B) 4-array stimuli used in the search tasks for the Target Averted Natural set (the 4-array stimuli were only used in the ‘search efficiency task’). (DOCX) [file pone.0228275.s005.docx]

**S2 Fig.**

| **Match** |  |
| --- | --- |
| **Dark** |  |
| **Light** |  |
| **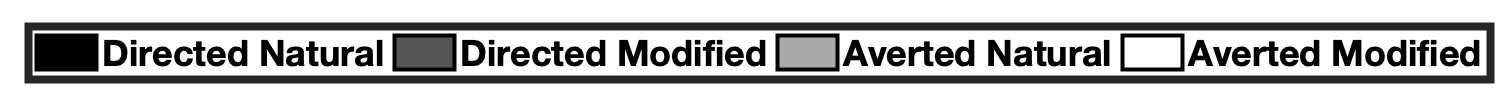** | |

Supplement: S2 Fig — Horizontal lines indicate planned comparisons that were statistically significant. (DOCX) [file pone.0228275.s006.docx]

**S3 Fig.**

| **Upright** |  |
| --- | --- |
| **Small** |  |
| **Inverted** |  |
| **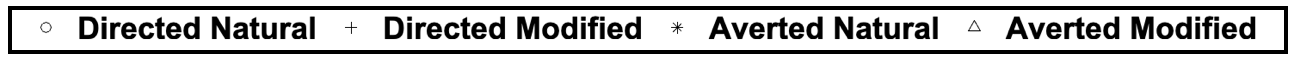** | |

Supplement: S3 Fig — (DOCX) [file pone.0228275.s007.docx]
